# Three-Dimensional Architecture of Glomerular Endothelial Cells Revealed by FIB-SEM Tomography

**DOI:** 10.3389/fcell.2021.653472

**Published:** 2021-03-11

**Authors:** Yuto Kawasaki, Yasue Hosoyamada, Takayuki Miyaki, Junji Yamaguchi, Soichiro Kakuta, Tatsuo Sakai, Koichiro Ichimura

**Affiliations:** ^1^Department of Anatomy and Life Structure, Juntendo University Graduate School of Medicine, Tokyo, Japan; ^2^Department of Nutrition, Faculty of Health Care Sciences, Chiba Prefectural University of Health Sciences, Chiba, Japan; ^3^Laboratory of Morphology and Image Analysis, Research Support Center, Juntendo University Graduate School of Medicine, Tokyo, Japan

**Keywords:** FIB-SEM tomography, block-face imaging, glomerular endothelial cell, mesangial cell, autocellular junction, 3D ultrastructure

## Abstract

Focused-ion beam-scanning electron microscopic (FIB-SEM) tomography enables easier acquisition of a series of ultrastructural, sectional images directly from resin-embedded biological samples. In this study, to clarify the three-dimensional (3D) architecture of glomerular endothelial cells (GEnCs) in adult rats, we manually extracted GEnCs from serial FIB-SEM images and reconstructed them on an Amira reconstruction software. The luminal and basal surface structures were clearly visualized in the reconstructed GEnCs, although only the luminal surface structures could be observed by conventional SEM. The luminal surface visualized via the reconstructed GEnCs was quite similar to that observed through conventional SEM, indicating that 3D reconstruction could be performed with high accuracy. Thus, we successfully described the 3D architecture of normal GEnCs in adult rats more clearly and precisely than ever before. The GEnCs were found to consist of three major subcellular compartments, namely, the cell body, cytoplasmic ridges, and sieve plates, in addition to two associated subcellular compartments, namely, the globular protrusions and reticular porous structures. Furthermore, most individual GEnCs made up a “seamless” tubular shape, and some of them formed an autocellular junction to make up a tubular shape. FIB-SEM tomography with reconstruction is a powerful approach to better understand the 3D architecture of GEnCs. Moreover, the morphological information revealed in this study will be valuable for the 3D pathologic evaluation of GEnCs in animal and human glomerular diseases and the structural analysis of developmental processes in the glomerular capillary system.

## Introduction

**FIGURE 1:**
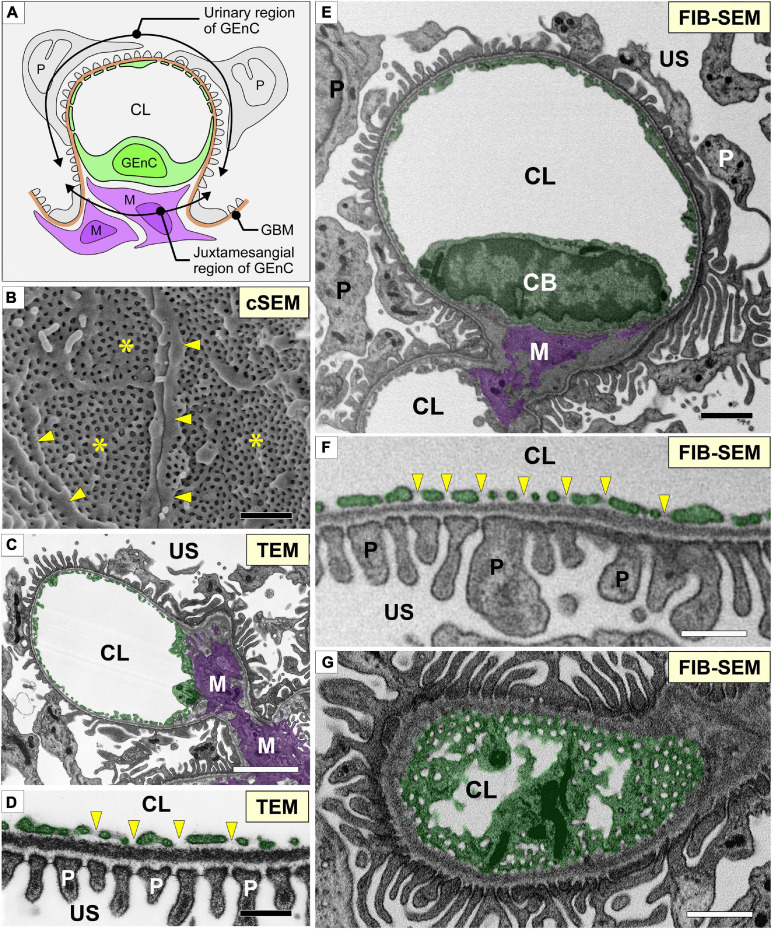
Glomerular capillary images of conventional electron microscopy and focused-ion beam-scanning electron microscopy (FIB-SEM). **(A)** Two regions of a glomerular endothelial cell (GEnC) in relation to the surrounding structures, such as the glomerular basement membrane (brown line, GBM), mesangial cells (purple cells, M), and podocytes (gray cells, P). **(B)** Conventional SEM (cSEM) image of the luminal surface of GEnCs. Highly fenestrated sieve plates (asterisks) and cytoplasmic ridges (arrowheads) are clearly visible. **(C,D)** Conventional TEM images. **(E–G)** Contrast-inverted FIB-SEM images. Conventional TEM and FIB-SEM images showing that the transverse sections of the glomerular capillary are similar in quality **(C–F)**. **(D,F)** Magnification of glomerular capillary wall, which consists of GEnCs, the GBM, and podocyte foot processes. The fenestrae of GEnCs do not exhibit diaphragms (arrowheads in **D,F**). **(G)** Grazing section of a capillary wall showing the fenestrae without diaphragms. CB, cell body of glomerular endothelial cell; CL, capillary lumen; US, urinary space. Scale bar, 5 μm in **(C,E,G)**; 200 nm in **(B,D,F)**.

Glomerular endothelial cells (GEnCs) form a glomerular capillary system between afferent and efferent arterioles (Kriz and Kaissling, 2000). The glomerular capillary system, together with the mesangium, creates a vascular compartment of the glomerulus. The vascular compartment is further enwrapped by an epithelial compartment, which is composed of the glomerular basement membrane (GBM) and podocytes, *en bloc* (Ichimura et al., 2007; Figure 1A). Vascular endothelial cells are structurally specialized to maintain the specific functions of individual organs. The relationship between their ultrastructure and permeability has been explored in many previous studies (Roberts and Palade, 2000). In the capillaries where the transendothelial exchange vigorously occurs, numerous transcellular pores or fenestrae form “sieve plates.” Such fenestrated endothelial cells are found in the exocrine and endocrine glands, liver sinusoid, intestinal villi, and kidney (glomerular and peritubular capillaries) (Pease, 1955; Maul, 1971). In general, endothelial fenestrae are furnished with fenestral diaphragms composed of a transmembrane glycoprotein, namely, PV-1 (Stan et al., 1999a, b; Stan et al., 2004). The fenestral diaphragms function by maintaining the diameters of these structures in a specific small range (Simionescu, 1983, 2000; Ichimura et al., 2008). For efficient glomerular ultrafiltration, the GEnCs cause fenestral diaphragms to disappear and enlarge the diameter during glomerular development (Reeves et al., 1980; Ichimura et al., 2008).

Endothelial cells face the lumen of the vasculature; thus, conventional scanning electron microscopy (SEM) is useful in evaluating their luminal surface structures. GEnCs can be exposed by freeze fracture and visualized by conventional SEM; however, only limited numbers and parts of GEnCs can be exposed by freeze fracture (Miyaki et al., 2020a). Thus, conventional SEM would not be enough to examine the whole architecture of individual GEnCs.

Focused-ion beam-SEM (FIB-SEM) tomography is a powerful approach to overcome this problem. This method of electron microscopy automatically acquires the serial sectional images directly from biological samples embedded in epoxy resin (Kubota, 2015; Ohno et al., 2015; Titze and Genoud, 2016). These FIB-SEM images are comparable to conventional transmission electron microscopy (TEM) images. Moreover, three-dimensional (3D) cellular structures can be reconstructed from serial sectional images with high reliability (Miyaki et al., 2020a, b). Recently, using this approach, our group has re-evaluated the 3D ultrastructure of podocytes in the context of health (Ichimura et al., 2015), development (Ichimura et al., 2017), disease (Ichimura et al., 2019), and evolution (Ichimura and Sakai, 2017; Kawasaki et al., 2019; Miyaki et al., 2020c). The 3D reconstructed images of single podocytes enable the evaluation of surface structures of these cells from any direction and without interference by neighboring podocytes and the GBM. Furthermore, other groups found that a direct interaction was formed between podocytes and mesangial cells in human glomerular diseases using serial block-face SEM, which is an alternative SEM method for acquiring the serial sectional images directly from resin-embedded tissue samples (Takaki et al., 2019; Nagai et al., 2020).

Focused-ion beam-SEM tomography with reconstruction techniques can also reveal the 3D architecture of nephron-constituent cells other than podocytes. In the present study, using FIB-SEM tomography and reconstruction techniques, we successfully described the 3D architecture of normal GEnCs in adult rats more clearly and precisely than ever before.

## Materials and Methods

### Animals

Two adult (10 weeks old, male) Wistar rats (Charles River Japan, Yokohama, Japan) were used in this study. Rats were perfused with physiological saline and subsequently 2.5% glutaraldehyde fixative buffered with 0.1 M phosphate buffer (PB) under anesthesia with pentobarbital. All procedures performed on laboratory animals were approved by the Institutional Animal Care and Use Committee of Juntendo University School of Medicine (approval no. 2020104). All animal experiments were carried out in accordance with the Guidelines for Animal Experimentation of Juntendo University School of Medicine.

### Conventional TEM

Conventional TEM was performed as described previously (Ichimura et al., 2009, 2010). In brief, the slices of fixed kidney cortex were successively immersed in 0.4% osmium tetroxide (OsO_4_) in 0.1 M PB for 1 h, 2% low-molecular-weight tannic acid (LMW-TA, Electron Microscopy Sciences, Hatfield, PA, United States) in 0.05 M maleate buffer for 4 h, and 1% uranyl acetate in 0.05 M maleate buffer for 3 h. The slices were then dehydrated with a graded series of ethanol and embedded in Oken Epok 812 epoxy resin (Oken-shoji, Tokyo, Japan). Ultrathin sections (90–100 nm thickness) were produced with an ultra 45° diamond knife (Diatome, Biel, Switzerland) and transferred to 50-mesh copper grids coated with a Formvar membrane. The ultrathin sections stained with lead citrate and uranyl acetate were digitally photographed with an H-7100 transmission electron microscope (Hitachi High-Technologies, Tokyo, Japan), which was equipped with a C4742-95 CCD camera (Hamamatsu Photonics, Shizuoka, Japan).

### Conventional SEM

Conventional SEM was performed as described previously (Dong et al., 2010; Miyaki et al., 2020a). Small cubes of the fixed kidney cortex (approximately 4 × 4 × 2 mm) were processed with conductive staining. First, the cubes were immersed in 1% OsO_4_ in 0.1 M PB for 30 min at 24°C, washed with 0.1 M PB for 5 min three times, and then immersed in 1% LMW-TA (Electron Microscopy Sciences) in distilled water (DW) for 2 h at RT. After the cubes were washed three times with DW for 5 min, the same staining procedure was repeated twice. However, OsO_4_ was diluted with DW.

The stained samples were dehydrated with a graded series of ethanol and then immersed in *t*-butyl alcohol. The samples were freeze-dried using an ES-2030 freeze dryer (Hitachi High-Technologies, Tokyo, Japan). The dried specimens were mounted on aluminum stubs with carbon paste (Pelco Colloidal Graphite, Ted Pella, Inc., Redding, CA, United States). The mounted specimens were coated with osmium with an OPC80T osmium plasma coater (Filgen, Inc., Nagoya, Japan). The samples were observed with an S-4800 field emission-SEM (Hitachi High-Technologies). Regions of interest were imaged using a backscattered electron detector with an acceleration voltage of 3 kV. Individual podocytes were denoted with different transparent colors using Adobe Illustrator.

### FIB-SEM Tomography

**FIGURE 2:**
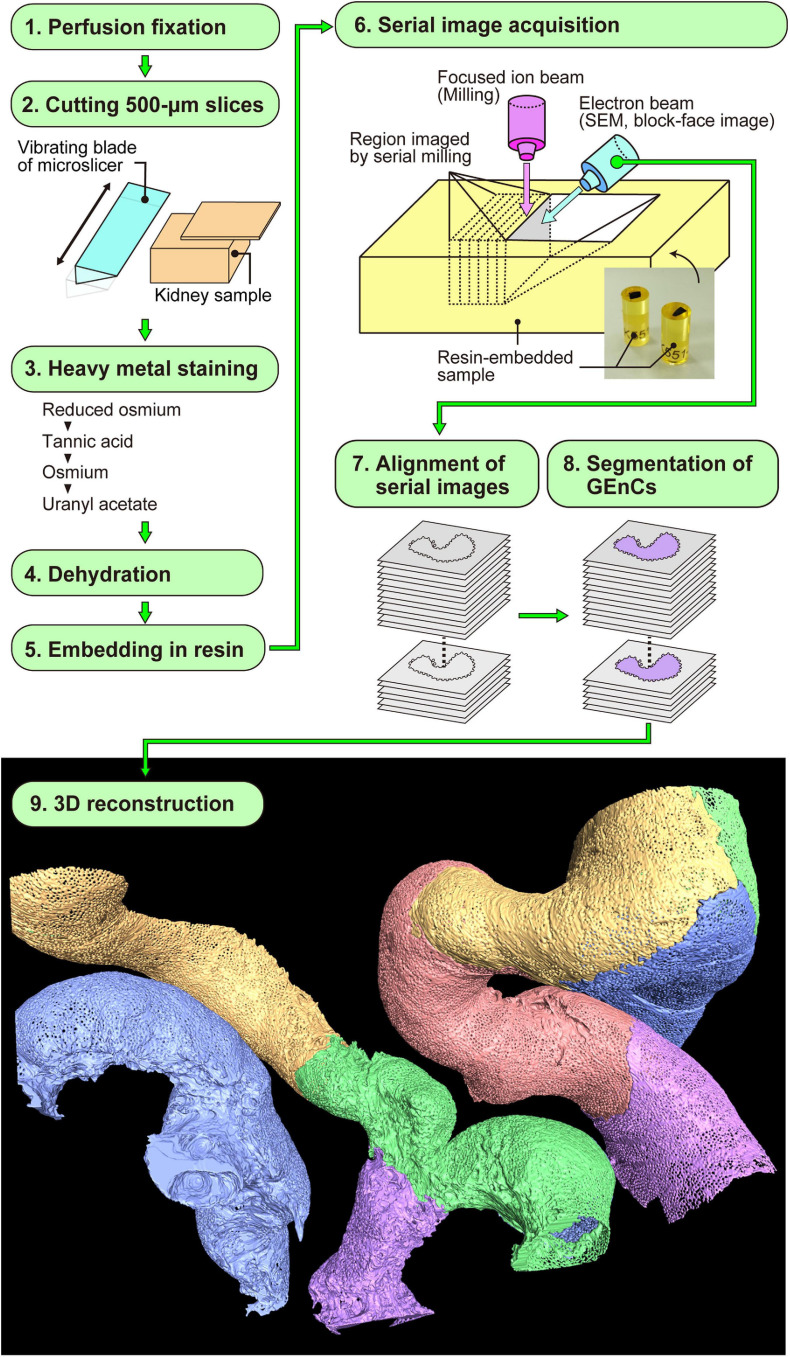
Focused-ion beam-scanning electron microscopy (FIB-SEM) tomography and 3D reconstruction of glomerular endothelial cells (GEnCs). The series of this method is largely divided into nine steps (1–9). Finally, the color-coded 3D reconstruction images of GEnCs are produced.

Focused-ion beam-SEM tomography was performed as described previously (Miyaki et al., 2020a, b; Figure 2). In brief, the cortex of the fixed kidney was cut into 250-μm-thick slices with a DTK-1000 MicroSlicers (Dosaka EM, Kyoto, Japan). The slices were successively immersed in 1% OsO_4_ containing 1.5% potassium ferrocyanide [K_4_Fe(CN)_6_⋅3H_2_O] in 0.1 M cacodylate buffer (CB) for 1 h on ice, 1% LMW-TA (Electron Microscopy Sciences) in 0.1 M CB for 4 h at 24°C, 2% aqueous OsO_4_ for 30 min at 24°C, and 1% aqueous uranyl acetate [UO_2_(CH_3_COO)_2_⋅2H_2_O] overnight at 24°C. The slices were washed with 0.1 M CB or DW for 5 min, three times, between each staining step.

The stained samples were then dehydrated using a graded series of ethanol. The dehydrated samples were successively immersed in propylene oxide for 5 min twice, 50% epoxy resin in propylene oxide on a rotational mixer for 3 h, and then pure epoxy resin on a rotational mixer for 12 h. Subsequently, the samples were embedded in newly made pure epoxy resin and hardened for 3 days at 60°C.

The surfaces of the resin-embedded tissues were exposed using a diamond knife on an Ultracut UCT (Leica Biosystems). The block was mounted onto an HV-8 aluminum stub (Micro Star, Tokyo, Japan). The space between the block and stub was filled with carbon paste and then coated with a thin layer of platinum–palladium, using an MC1000 ion sputter coater (Hitachi High-Technologies).

The coated surface of the block was imaged with a Helios Nanolab 660 FIB-SEM (Thermo Fisher Scientific, Waltham, MA, United States), at a high acceleration voltage of 10 kV, to determine the area of interest. To prevent beam damage, a platinum layer was deposited on the area of interest, with a 2.5 nA beam current, where a voltage of 30 kV accelerated gallium ions. To make the new block face (imaging-face), a trench was cut using FIB milling, with a 25 nA beam current, where gallium ions were accelerated by a voltage of 30 kV. Serial FIB-SEM images were acquired with Auto Slice and View imaging software (Thermo Fisher Scientific) on a Helios Nanolab 660 FIB-SEM. New surfaces for serial block-face imaging were generated using FIB milling, with a 0.77 nA beam current. Three sets of serial 850 FIB-SEM images were obtained from two adult rats every 50 nm in depth, with a backscattered electron detector at an acceleration voltage of 3.0 kV. The size of each FIB-SEM image was 41.5 × 27.7 μm (width × height). Thus, the volume of serial image acquisition was 41.5 × 27.7 × 42.5 μm (width × height × depth).

### Three-Dimensional Reconstruction

After aligning serial FIB-SEM images, we manually segmented the target GEnCs on individual FIB-SEM images using AMIRA 6.1 reconstruction software (Thermo Fisher Scientific) and then reconstructed them using the same software (Figure 2). We used a Cintiq 27QHD large interactive pen display for the segmentation procedure (Wacom, Tokyo, Japan).

## Results

### Sectional FIB-SEM Images of GEnCs

We obtained serial FIB-SEM images of the glomeruli of adult Wistar rats. The contrast-inverted FIB-SEM images of the glomerulus achieved a quality comparable to that of conventional TEM images (Figures 1C,D,E–G). In spatial relation to the surrounding structures, the glomerular capillary and GEnCs were divided into two regions, urinary and juxtamesangial regions, as previously proposed by Sakai and Kriz (1987; Figure 1A). The urinary region made up 70–80% of the total circumference in a GEnC and adhered to the GBM (Figures 1E,F). The urinary region was also highly attenuated and fenestrated (Figures 1F,G). The fenestrae in GEnCs were not furnished with fenestral diaphragms, unlike that in other fenestrated endothelial cells, such as peritubular capillary endothelial cells (arrows in Figure 1F). The juxtamesangial region made up 20–30% of the total circumference of a GEnC and was in contact with the mesangium without the GBM between them (Figures 1A,E). The cell bodies of GEnCs, which contained a nucleus and bulged into the capillary lumen, were usually localized at the juxtamesangial region (Figures 1A,E).

### Three-Dimensional Architecture of GEnCs Revealed by 3D Reconstruction

**FIGURE 3:**
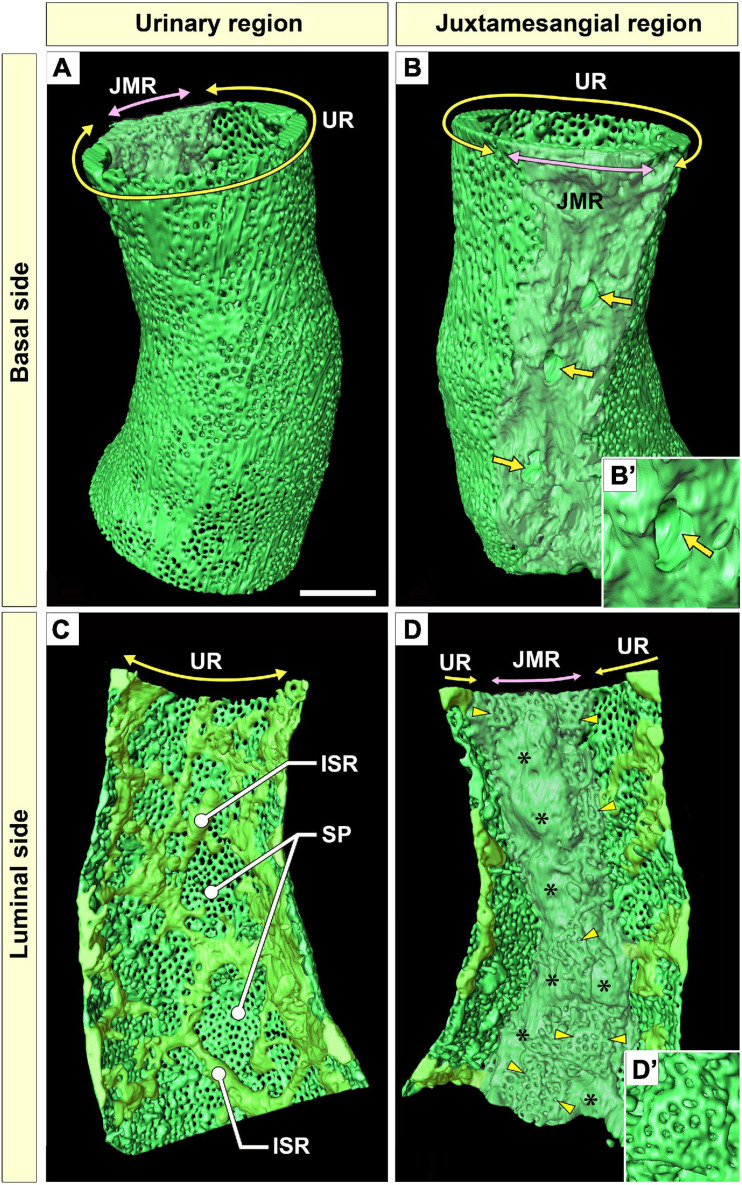
Urinary region (UR) and juxtamesangial region (JMR) of a glomerular endothelial cell (GEnC). **(A,B)** Three-dimensional reconstruction of a single GEnC tube. The basal surfaces of the UR and JMR are predominantly shown in **(A,B)**, respectively. **(A)** In the UR, the basal surface is smooth in total despite the high fenestration. **(B)** In the JMR, the basal surface is uneven with less fenestration. The indentations for mesangial cell processes were frequently recognized (arrows). **(B′)** High magnification of an indentation created by a large non-adhesive mesangial cell process. The GEnC tube in **(A,B)** is longitudinally divided into two halves, and the luminal surfaces of the UR **(C)** and JMR **(D)** are shown. **(C)** In the UR, the sieve plates are among the fine intersieve ridges (yellowish-green). **(D)** In the JMR, the thick juxtamesangial cytoplasmic ridge could be found (asterisks, whitish-green). The reticular porous structures are frequently recognized on and beside the thick juxtamesangial ridge (arrowheads). **(D′)** High magnification of the reticular porous structure. ISR, intersieve ridge; SP, sieve plate. Scale bar, 5 μm. The reconstructed images of GEnC **(A–D)** are also shown in Supplementary Movies 1–3.

**FIGURE 4:**
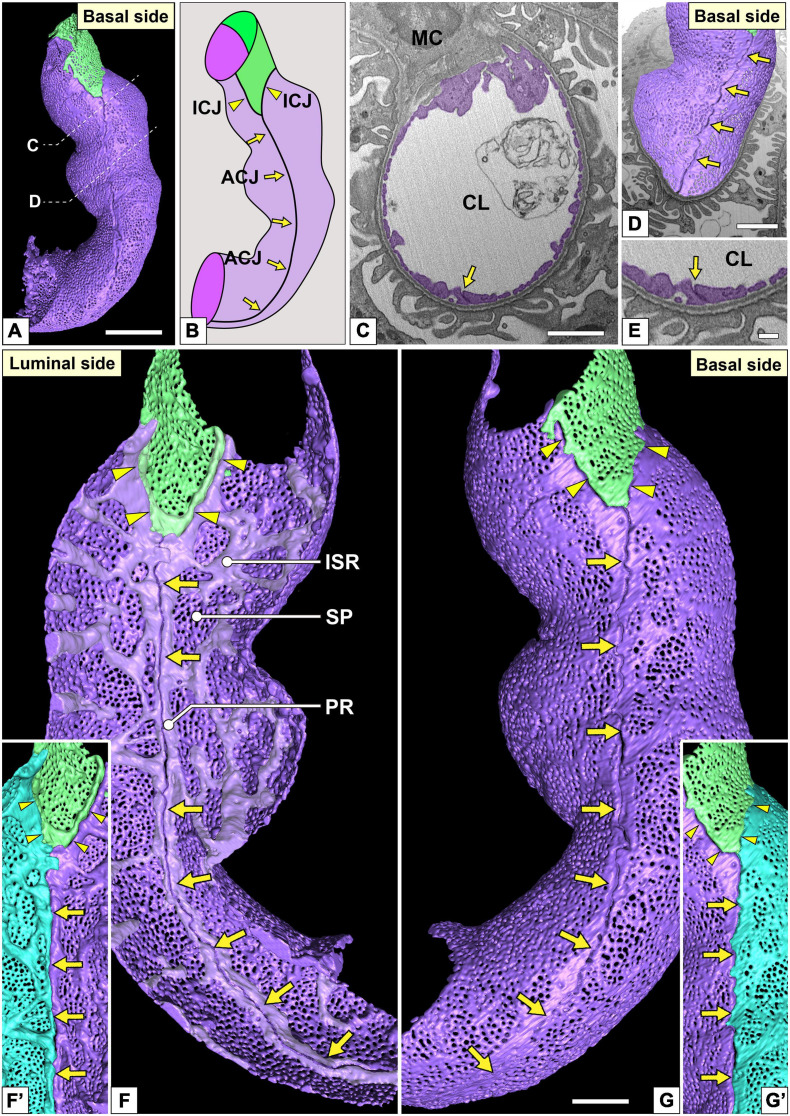
Urinary region and autocellular junction of glomerular endothelial cells (GEnCs). **(A)** A reconstructed capillary is formed by two GEnCs (purple and green). **(B)** The location of cellular junctions in this capillary. The purple GEnC forms an autocellular junction (ACJ), which is along the longitudinal axis of the capillary. ICJ, intercellular junction between green and purple GEnCs. **(C)** The sectional focused-ion beam-scanning electron microscopy (FIB-SEM) image of the purple GEnC. CL, capillary lumen; MC, mesangial cell. **(D)** The reconstructed GEnCs are displayed on the sectional FIB-SEM image. **(E)** Magnified image of the ACJ shown in **(C)**. The luminal **(F,F′)** and basal **(G,G′)** surfaces of the urinary region. **(F)** Two peripheral ridges of the purple GEnC bulge into the capillary lumen and are aligned in parallel to form an ACJ (arrows in **F**). Between purple and green GEnCs, an intercellular junction is formed via their peripheral ridges (arrowheads in **F**). The peripheral and intersieve ridges were represented by whitish-purple. **(G)** The basal surface of these cytoplasmic ridges is smooth. **(F′,G′)** To show the location of the ACJ more clearly, a part of the purple GEnC is displayed on the right in blue. Arrows, ACJ; arrowheads, ICJ. ISR, intersieve ridge; PR, peripheral ridge; SP, sieve plate. Scale bar, 5 μm in **(A,C,D)**; 2 μm in **(G)**; and 100 nm in **(E)**. The reconstructed image of GEnCs **(F,G)** is also shown in Supplementary Movie 4.

**FIGURE 5:**
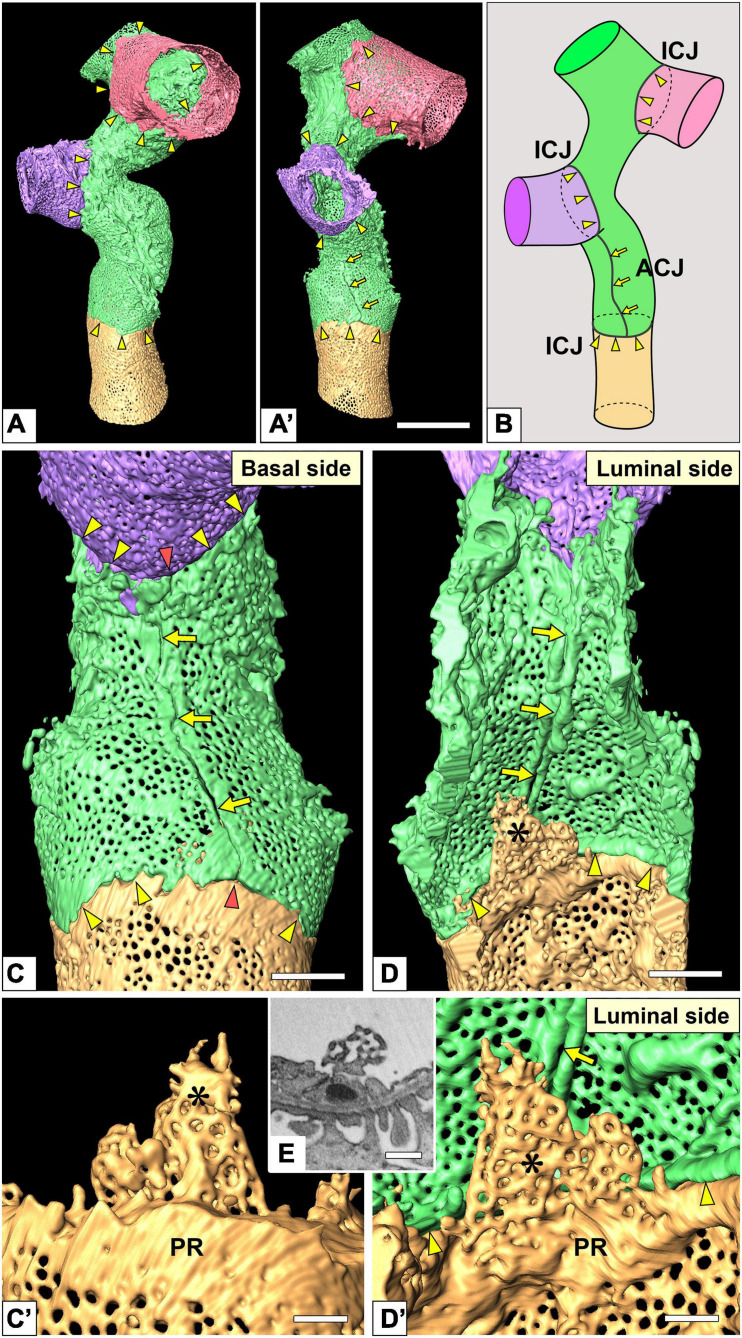
Intercellular junction and autocellular junction (ACJ) of glomerular endothelial cells (GEnCs). **(A)** Reconstruction of a branching capillary formed by four GEnCs (purple, green, red, and yellow). **(A′)** View of the capillary shown in **(A)** from a different angle. **(B)** The location of cellular junctions in this capillary. The green GEnC forms an ACJ, which is along the longitudinal axis of the capillary. ICJ, intercellular junction between adjacent GEnCs. The basal **(C)** and luminal **(D)** surfaces of the urinary region. Two peripheral cytoplasmic ridges of the green GEnC are aligned in parallel to form an ACJ (arrows). The ACJ meets the ICJ to create “inverted T-shaped” seams (red arrowheads). Yellow arrowheads, ICJs between green and yellow GEnCs. **(C′,D′)** Magnified image of a reticular porous structure formed on the peripheral ridge (PR) of the yellow GEnC. **(C′)** The green GEnC is removed from **(C)**. **(E)** Focused-ion beam-scanning electron microscopy (FIB-SEM) image of the reticular porous structure shown in **(C′,D′)**. Asterisks, reticular porous structure. Scale bar, 10 μm in **(A′)**; 2 μm in **(C,D)**; 100 nm in **(C′,D′,E)**. The reconstructed images of GEnCs **(A,A’,C,D)** are also shown in Supplementary Movies 5, 6.

We reconstructed six glomerular capillary loops containing 24 GEnCs from serial FIB-SEM images (Figure 2). Both the luminal and basal surface structures were clearly visualized in the reconstructed GEnCs (Figures 3–5 and Supplementary Movies 1–6), although only the luminal surface structures could be observed by conventional SEM (Figure 1B). The luminal surface visualized based on the reconstructed GEnCs was quite similar to that observed by conventional SEM (Figures 1B, 3C, 4F), indicating that 3D reconstruction could be performed with high accuracy.

#### Urinary Region

**FIGURE 6:**
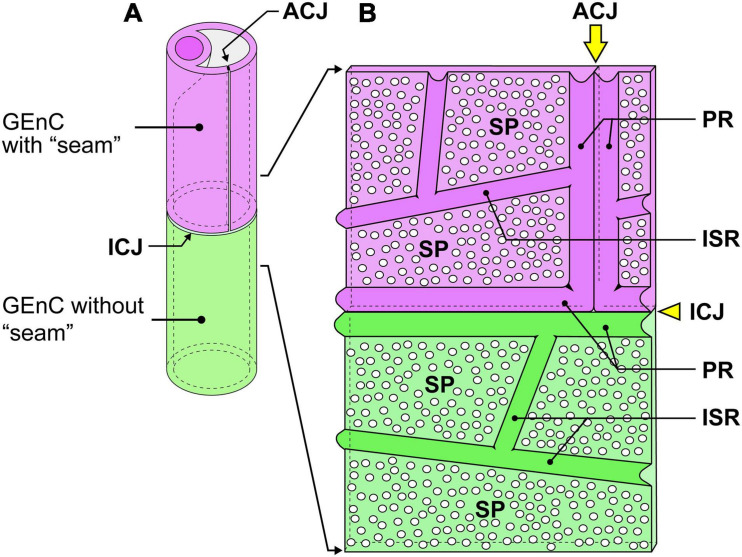
Schematic diagram of the cytoplasmic ridges and cellular junctions in the urinary region. **(A)** A green glomerular endothelial cell (GEnC) forms a capillary tube without an autocellular tight junction. This type of capillary is the so-called “seamless capillary”; a purple GEnC makes an autocellular junction (ACJ) to form a capillary tube. The ACJ is usually along the longitudinal axis of the capillary. Two GEnCs are connected by an intercellular junction (ICJ), which is usually aligned perpendicularly to the longitudinal axis of the capillary. **(B)** Luminal view of the adhering region between purple and green GEnCs. The capillary tube shown in **(A)** is cut lengthwise and unfolded. The peripheral ridge (PR) forms the cell periphery of GEnCs; thus, two PRs of adjacent GEnCs are parallel. The intersieve ridge (ISR) is between the sieve plates (SPs), which are highly attenuated and fenestrated.

**FIGURE 7:**
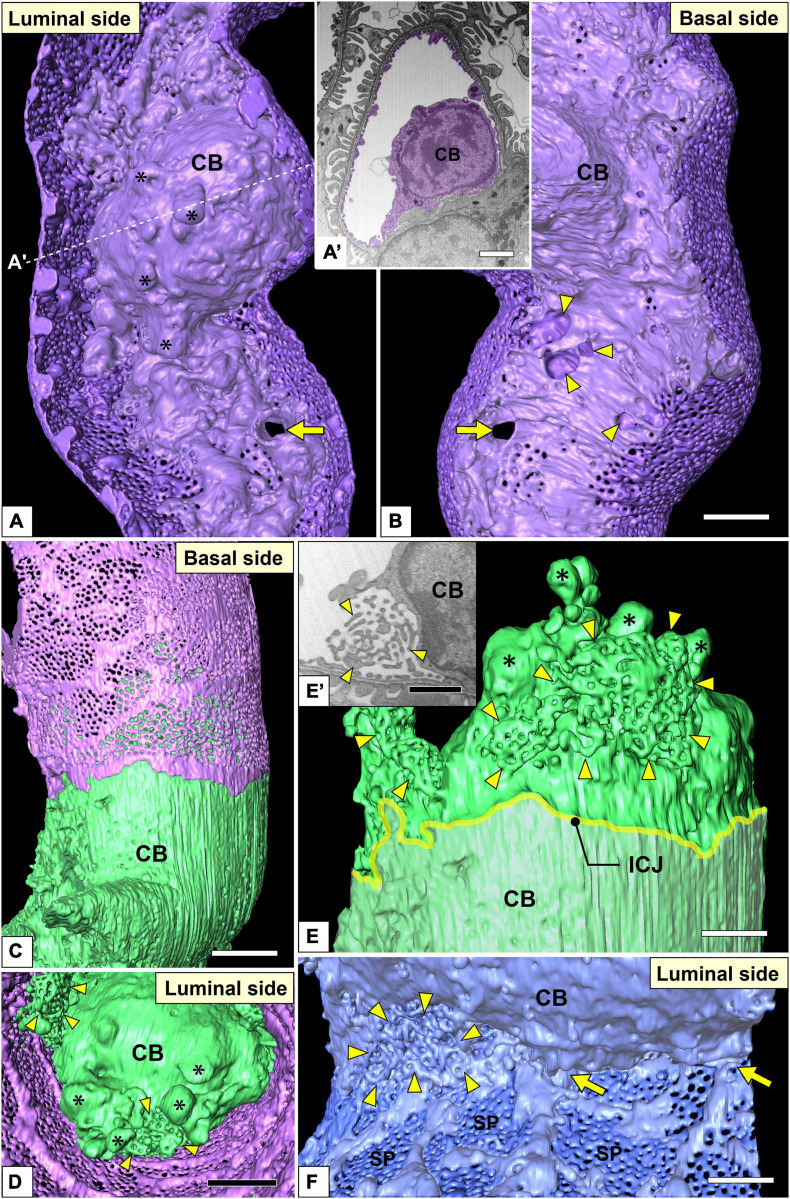
Juxtamesangial region and cell bodies of glomerular endothelial cells (GEnCs). Luminal **(A)** and basal **(B)** views of the same juxtamesangial region. The globular protrusions (asterisks) are found on the luminal surface of the cell body. The non-adhesive mesangial cell processes create the perforation (arrow) and indentations (arrowheads) at the juxtamesangial region. **(A′)** The sectional focused-ion beam-scanning electron microscopy (FIB-SEM) image at the dotted line in **(A)**. **(C–E)** Cell body of the green GEnC. This cell body possesses reticular porous structures (arrowheads in **D,E**), in addition to globular protrusions (asterisks in **D,E**), on the luminal surface. **(E)** The purple GEnC is removed from **(C)** to show the luminal surface of the green GEnC. **(E′)** FIB-SEM image of the reticular porous structure shown in **(E)**. **(F)** Luminal view of the connecting part of urinary and juxtamesangial regions. The intersieve ridges continue the cell body (arrows), and the reticular porous structure (arrowheads) exists at the angle between urinary and juxtamesangial regions. ICJ, intercellular junction between green and purple GEnCs; CB, cell body of GEnC; SP, sieve plate. Scale bar, 5 μm in **(A′,B,D)**; 2 μm in **(E,F)**; 100 nm in **(E′)**. The reconstructed image of GEnC **(A,B)** is also shown in Supplementary Movie 7.

The urinary regions of GEnCs were highly attenuated and fenestrated (Figures 3A,C and Supplementary Movies 1, 2). The basal surface of the urinary region was smooth since it adhered to the distended, smooth GBM (Figure 3A and Supplementary Movie 1). The urinary region was subdivided into sieve plates and cytoplasmic ridges. The sieve plates exhibited numerous dense fenestrae (Figures 3C, 4G and Supplementary Movies 2, 4), and the cytoplasmic ridges were formed between the sieve plates (as intersieve ridges; asterisks in Figures 3C, 4G). The cytoplasmic ridges were also formed along the cell margin (as a peripheral ridge; arrowheads in Figures 4F,F′, 5D and Supplementary Movie 4), and the GEnCs connected at their peripheral ridges via a tight junction (Figures 4C–E, 6B); thus, the two peripheral ridges ran parallel to each other (the cellular junctions of GEnCs will be described in detail later). The intersieve ridges usually continued to the peripheral ridge (Figure 6B) and cell body (Figure 7F). The reticular porous structures were frequently found on the cytoplasmic ridges, especially on the peripheral ridges (asterisks in Figures 5C′,D,D′).

#### Juxtamesangial Region

**FIGURE 8:**
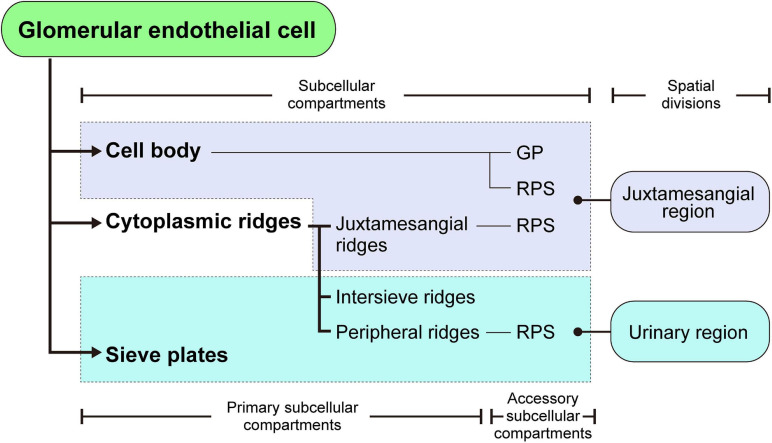
Structural hierarchy of the subcellular compartments in a glomerular endothelial cell (GEnC). The reticular porous structures (RPSs) are formed on the cell body, juxtamesangial ridges, and peripheral ridges, with the globular protrusions (GPs) on the cell body.

The juxtamesangial region was less fenestrated (Figures 3B,D and Supplementary Movies 1, 3). This region consisted of the cell body (Figures 7B,D and Supplementary Movie 7) and thick cytoplasmic ridges, referred to as juxtamesangial ridges (asterisks in Figure 3D and Supplementary Movie 3). The reticular porous structures were frequently found on the luminal surfaces of the cell bodies (arrowheads in Figures 7C–E,E′) and the juxtamesangial ridges (arrowheads in Figures 3D,D′). Moreover, in some GEnCs, globular protrusions were also found on the luminal surface of the cell bodies (asterisks in Figures 7A,A′,D,E and Supplementary Movie 7). The spatial arrangement of subcellular compartments was thus revealed in GEnCs (as summarized in Figure 8).

**FIGURE 9:**
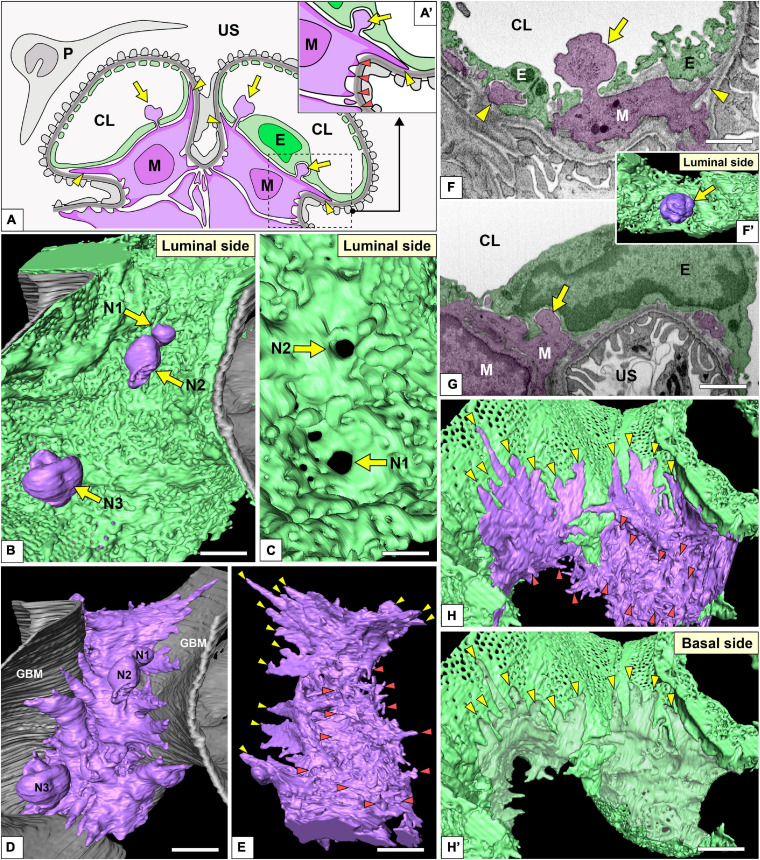
Spatial relationship among glomerular endothelial cells (GEnCs) and mesangial cells. **(A,A′)** Schematic diagram showing the two-dimensional geometry of GEnCs (green, E), mesangial cells (purple, M), podocytes (gray, P), and the glomerular basement membrane (GBM). The non-adhesive mesangial cell processes (arrows) protrude toward the juxtamesangial region of the GEnC to form the indentation or penetrate it into the capillary lumen. Most of the adhesive mesangial cell processes are spiny cytoplasmic processes and adhere to the GBM at their tips (orange arrowheads in **A′**). Some adhesive mesangial cell processes exhibit large, tongue-like shapes and are inserted into the narrow angle between the GEnC and GBM (yellow arrowheads in **A,A′**). **(B)** Luminal view of three non-adhesive mesangial cell processes (purple, N1–N3) penetrating the green GEnC. Gray layer, GBM. **(C)** The large holes of GEnCs (arrows) for the penetration of mesangial cell processes, N1 and N2. **(D)** The spatial relationship between the mesangial cell and GBM. The green GEnC shown in **(B)** is removed to reveal the mesangial cell processes. **(E)** The mesangial cells shown in **(D)** are rotated 180° to show the spiny mesangial cell processes (orange arrowheads). Some of them develop tongue-like projections (yellow arrowheads). **(F,G)** Focused-ion beam-scanning electron microscopy (FIB-SEM) images of adhesive (arrowheads) and non-adhesive mesangial cell processes (arrows). **(F′)** Three-dimensional reconstruction of the non-adhesive process shown in **(F)**. **(H,H′)** The impression created by the tongue-like processes against the basal surface of the juxtaglomerular region (yellow arrowheads in **H′**). CL, capillary lumen; US, urinary space. Scale bar, 2 μm in **(B,D,E,H′)**; 200 nm in **(C,F,G)**. The reconstructed image of GEnC and mesangial cell **(B,E)** is also shown in Supplementary Movie 8.

On the basal surface of the juxtamesangial region, indentations created by mesangial cell processes were frequently recognized (arrows in Figures 3B, 7B, 9G; arrowheads in Figure 9H′). Mesangial cells showed the extension of numerous cytoplasmic processes, which were classified into two groups, adhesive and non-adhesive. The adhesive processes adhered to the GBM at their tips (red and yellow arrowheads in Figures 9A,A′); the non-adhesive processes extend toward the juxtamesangial regions of GEnCs (arrows in Figures 9A,A′).

Most of the adhesive processes were tiny spines based on their shapes (red arrowheads in Figures 9A′,D–H and Supplementary Movie 8). Some of the adhesive processes exhibited large, tongue-like shapes and were inserted into the narrow angle between the GEnC and GBM (yellow arrowheads in Figures 9A,E,H). The indentations created by the tongue-like processes were found on the basal surface of the juxtaglomerular region (yellow arrowheads in Figure 9H′).

The non-adhesive processes protruded toward the basal surface of the cell body and juxtamesangial ridges to make deep indentations on them (arrows in Figures 3B, 7B, 9G and Supplementary Movie 8). Some non-adhesive processes penetrated the juxtamesangial ridges to enter the capillary lumen (arrows in Figures 9A–C,F,F′). These penetrating non-adhesive processes were irregularly enlarged in the capillary lumen.

#### Cellular Junctions

Most of the individual GEnCs (20 of total 24 reconstructed GEnCs) were recognized to form a tubular shape, except for four GEnCs. In 17 of the 20 total tubular GEnCs, the individual GEnCs formed a tubular shape without a seam—such endothelial cell types are referred as “seamless endothelial cells” (green cell in Figure 6A; Wolff and Bär, 1972; Bär et al., 1984). The adjacent seamless GEnCs were connected in an end-to-end manner via the intercellular junction, structurally regarded as a tight junction. The intercellular junctions between GEnCs were generally aligned circumferentially to the longitudinal axis of the glomerular capillary (Figures 5A,A′,B and Supplementary Movie 5). At the bifurcation area of the glomerular capillary, two tubular GEnCs were typically connected in an end-to-side manner (Figures 5A,A′).

Three of the 20 total tubular GEnCs formed a tubular shape by forming an autocellular junction (purple cell in Figure 6A), which was structurally regarded as a tight junction similar to the intercellular junction (arrow in Figure 5E). These autocellular junctions were generally along the longitudinal axis of the capillary (arrows in Figures 4, 5). They continued to the circumferential intercellular junction to form a “T-shaped” seam (red arrowheads in Figure 5C).

## Discussion

### Benefits of FIB-SEM Tomography for Structural Analysis of GEnCs

The 3D ultrastructures observed on the reconstructed GEnCs based on serial FIB-SEM images could be confirmed via conventional SEM images, indicating that the reconstructed images have high accuracy. This highly accurate visibility of the 3D ultrastructures is one of the most important benefits of this approach. Conventional SEM is useful for revealing the luminal surface structures of GEnCs; however, it is almost impossible to observe their basal surface structures because the basal surface is attached to the GBM or mesangium and is difficult to expose. The 3D reconstruction ultimately resolved this problem by enabling the observation of surface structures from any direction and without interference by the neighboring GBM, mesangium, and podocytes. Furthermore, the color-coded reconstructed images are also helpful in determining the spatial relationship between GEnCs and mesangial cells, for example, the arrangement of GEnC intercellular and autocellular junctions and the penetration of mesangial non-adhesive processes into the glomerular capillary lumen.

### Definition and Structural Hierarchy of Subcellular Compartments in GEnCs

The 3D reconstruction of GEnCs is also helpful in determining the structural hierarchy of their subcellular compartments (as summarized in Figure 8). The GEnCs were largely divided into three kinds of primary subcellular compartment: cell body, cytoplasmic ridge, and sieve plate. The cell body contains a nucleus and bulged toward the capillary lumen. The cytoplasmic ridges form a net that exhibits coarse and irregular-shaped meshes around the cell body. The sieve plates, which are surrounded by the cytoplasmic ridges, are highly fenestrated regions for glomerular filtration. Based on the location of the presence, the cytoplasmic ridges are further divided into the juxtamesangial, intersieve, and peripheral ridges. In addition to these primary subcellular compartments, two accessory subcellular compartments, the reticular porous structure and globular protrusion, are frequently associated with the cell body and cytoplasmic ridges.

### Characteristics of Cellular Junctions in GEnCs

Individual GEnCs generally make up the entire circumference of a capillary tube. Most of the individual GEnCs form a capillary tube without an autocellular junction (green GEnC in Figure 6A)—such endothelial cells have been called “seamless endothelial cells” (Wolff and Bär, 1972; Bär et al., 1984). Some GEnCs formed an autocellular junction to form a capillary tube (purple GEnC in Figure 6A). During the developmental remodeling of the capillary system in zebrafish larvae, the endothelial cells lose their autocellular junction to self-fuse and transform into a seamless endothelial cell (Lenard et al., 2015). Thus, the seamless GEnC is likely formed from the GEnC with an autocellular junction via self-fusion. However, to understand the developmental process of seamless GEnCs, it is necessary to analyze the developing glomerular capillaries in perinatal animals.

### Relationship Between GEnCs and Mesangial Cells

A higher blood pressure (approximately 50 mm Hg) is loaded in the glomerular capillary, which is a driving force of glomerular ultrafiltration. To protect the glomerular structure against its higher intracapillary pressure, several mechanical protectors are supported in both the vascular and epithelial compartments (Ichimura et al., 2007; Ichimura and Sakai, 2017). Mesangial cells, mechanical protectors, adhere to the internal surface of the GBM through their adhesive processes and pull centripetally to prevent overextension of the epithelial component (Sakai and Kriz, 1987; Drenckhahn et al., 1990).

Farquhar and Palade (1962) previously reported the mesangial cell processes entering the capillary lumen, which corresponded to the non-adhesive processes presented in this study. The phenomenon that mesangial cell processes penetrate GEnCs has not been examined beyond this study. The non-adhesive mesangial cell processes were prominently enlarged at the end; thus, they were frequently found in the sectional FIB-SEM images as cytoplasmic masses. However, it is difficult to determine whether such cytoplasmic masses are derived from mesangial cells through conventional SEM and TEM, because the penetrating part of the mesangial cell is thin. This is the reason why research on this phenomenon has not proceeded. The functions of the non-adhesive processes of mesangial cells are unknown at this time, although Farquhar and Palade (1962) speculated that these mesangial cell processes serve as a sensor for the capillary lumen environment.

### Further Adaptation of FIB-SEM Tomography to GEnC Research

Focused-ion beam-SEM tomography with a 3D reconstruction technique is a powerful approach used in our previous podocyte research (Ichimura et al., 2015, 2017, 2019; Kawasaki et al., 2019; Miyaki et al., 2020c). In the present study, we clearly visualized the 3D cytoarchitecture of normal GEnCs in adult rats. However, as in podocytes, this approach should help in the 3D ultrastructural analysis of GEnCs under developmental and pathological conditions.

The development of the glomerular capillary system starts from the S-shaped body stage of glomerulogenesis (Reeves et al., 1978; Ichimura et al., 2003). The growth process of the glomerular capillary system is considered to be mediated by two types of angiogenesis processes, namely, sprouting angiogenesis and intussusceptive angiogenesis. In the sprouting process, the newly formed capillary is branched from the existing capillary; in the intussusceptive process, the existing capillary is split into two daughter capillaries (Patan et al., 1992; Djonov et al., 2000; Burri et al., 2004). However, it remains unknown as to which of the two angiogenesis processes is used to develop the glomerular capillary system. Our group is currently evaluating the morphological development of the glomerular capillary system using FIB-SEM tomography and 3D reconstruction techniques and will soon report our results.

### Limitations of 3D Ultrastructural Analysis Using FIB-SEM Tomography

Focused-ion beam-SEM tomography could precisely reveal the 3D architecture of GEnCs; however, there are several technical disadvantages to this approach. First, image acquisition is limited to a relatively small volume in FIB-SEM tomography; thus, it is quite challenging to obtain the total volume of a glomerulus at a high resolution to evaluate the delicate structures of GEnCs. Owing to this problem, it was impossible to analyze the entire architecture of the glomerular capillary system. Array tomography is a solution to this problem. It has been used to obtain serial images from serial ultrathin sections made by a diamond knife with specimens mounted on a base substrate, such as silicon warfare or a glass plate (Micheva and Smith, 2007; Dittmayer et al., 2018). The serial ultrathin sections on the base substrate are physically stable and repeatedly observable by SEM. Terasaki et al. (2020) visualized the 3D architecture of a normal glomerular capillary system by array tomography using an ATUMtome, which automatically collects serial ultrathin sections on a tape base (Hayworth et al., 2014). This method will be useful for evaluating 3D pathologic alterations of the glomerular capillary system, such as the hilar neovascularization in diabetic nephropathy (Min and Yamanaka, 1993; Osterby et al., 1999) and the reorganization of the capillary system in mesangioproliferative glomerulonephritis (Kriz et al., 2003; Notoya et al., 2003; Ichimura et al., 2006).

## Conclusion

Focused-ion beam-SEM tomography was able to clearly visualize the 3D architecture of normal GEnCs. The morphological information revealed in this study will be valuable for 3D pathologic analyses of GEnCs in animal and human glomerular diseases.

## Data Availability Statement

The raw data supporting the conclusions of this article will be made available by the authors, without undue reservation.

## Ethics Statement

The animal study was reviewed and approved by the Institutional Animal Care and Use Committee of Juntendo University School of Medicine (approval no. 2020104).

## Author Contributions

KI designed the experiments, prepared the figures, and wrote the manuscript. YK, TM, JY, SK, and KI obtained serial FIB-SEM images. YK, YH, and KI performed 3D reconstruction. YK, YH, TS, and KI analyzed the experimental data. All authors contributed to the article and approved the submitted version.

## Conflict of Interest

The authors declare that the research was conducted in the absence of any commercial or financial relationships that could be construed as a potential conflict of interest.
