# Supplementary material for: Three-Dimensional Architecture of Glomerular Endothelial Cells Revealed by FIB-SEM Tomography
Source: Front Cell Dev Biol. 2021 Mar 11;9:653472. doi: 10.3389/fcell.2021.653472 (PMC7991748; doi:10.3389/fcell.2021.653472)
Supplement: Supplementary file 9 [file Data_Sheet_1.DOCX]

Supplementary Material

# Supplementary Movies

**Supplementary Movie 1.** The reconstructed capillary tube shown in **Fig. 3A, B** is rotated around the vertical axis.

**Supplementary Movie 2.** The urinary region of the reconstructed capillary tube shown in **Fig. 3C** is rotated around the vertical axis.

**Supplementary Movie 3.** The juxtamesangial region of the reconstructed capillary tube shown in **Fig. 3D** is rotated around the vertical axis.

**Supplementary Movie 4.** The urinary region of reconstructed capillary tube shown in **Fig. 4F, G** is rotated around the vertical axis.

**Supplementary Movie 5.** The reconstructed capillary tube shown in **Fig. 5A, Aʹ** is rotated around the vertical axis.

**Supplementary Movie 6.** The urinary region of the reconstructed capillary tube shown in **Fig. 5C, D** is rotated around the vertical axis.

**Supplementary Movie 7.** The juxtamesangial region of the reconstructed capillary tube shown in **Fig. 7A, B** is rotated around the vertical axis.

**Supplementary Movie 8.** The reconstructed capillary tube and associated mesangial cell shown in **Fig. 9B, E** is rotated around the vertical axis.
